# The Genomics and Metagenomics of Asthma Severity (GEMAS) Study: Rationale and Design

**DOI:** 10.3390/jpm10030123

**Published:** 2020-09-11

**Authors:** Javier Perez-Garcia, José M. Hernández-Pérez, Ruperto González-Pérez, Olaia Sardón, Elena Martin-Gonzalez, Antonio Espuela-Ortiz, Elena Mederos-Luis, Ariel Callero, Esther Herrera-Luis, Paula Corcuera, Inmaculada Sánchez-Machín, Paloma Poza-Guedes, Luis Manuel González García, Purificación Ramírez-Martín, Lorenzo Pérez-Negrín, Hemily Izaguirre-Flores, Javier Barrios-Recio, Eva Pérez-Rodríguez, Julia Alcoba-Florez, José A. Cañas, José M. Rodrigo Muñoz, Victoria del Pozo, Javier Korta-Murua, Lina I. Pérez Méndez, Mariano Hernandez-Ferrer, Jesús Villar, Fabian Lorenzo-Diaz, Maria Pino-Yanes

**Affiliations:** 1 Genomics and Health Group, Department of Biochemistry, Microbiology, Cell Biology and Genetics, Universidad de La Laguna, 38200 San Cristóbal de La Laguna, Santa Cruz de Tenerife, Spain; jpegarci@ull.edu.es (J.P.-G.); alu0101015408@ull.edu.es (E.M.-G.); aespuela@ull.edu.es (A.E.-O.); eherrerl@ull.edu.es (E.H.-L.); florenzo@ull.edu.es (F.L.-D.); 2 Pulmonary Medicine Service, Hospital Universitario de N.S de Candelaria, 38010 Santa Cruz de Tenerife, Spain; jmherper@hotmail.com (J.M.H.-P.); pramirez_martin@hotmail.com (P.R.-M.); lperneg@gobiernodecanarias.org (L.P.-N.); 3 Pulmonary Medicine Section, Hospital General de La Palma, 38713 Breña alta, Santa Cruz de Tenerife, Spain; lgongarc@gmail.com; 4 Allergy Department, Hospital Universitario de Canarias, 38320 San Cristóbal de La Laguna, Santa Cruz de Tenerife, Spain; glezruperto@gmail.com (R.G.-P.); elenamederosluis@gmail.com (E.M.-L.); zerupean67@gmail.com (I.S.-M.); pozagdes@hotmail.com (P.P.-G.); 5 Severe Asthma Unit, Allergy Department, Hospital Universitario de Canarias, 38320 San Cristóbal de La Laguna, Santa Cruz de Tenerife, Spain; 6 Division of Pediatric Respiratory Medicine, Hospital Universitario Donostia, 20014 San Sebastián, Spain; OLAIA.SARDONPRADO@osakidetza.eus (O.S.); PAULA.CORCUERAELOSEGUI@osakidetza.eus (P.C.); JOSEJAVIER.KORTAMURUA@osakidetza.eus (J.K.-M.); 7 Department of Pediatrics, University of the Basque Country (UPV/EHU), 20014 San Sebastián, Spain; 8 Allergy Unit, Hospital Universitario N.S. de Candelaria, 38010 Santa Cruz de Tenerife, Spain; arielcallero@hotmail.com (A.C.); javierbarrios_17@hotmail.com (J.B.-R.); evaprod@hotmail.com (E.P.-R.); 9 Breña Baja Health Center, Health Area of La Palma, 38711 Breña Baja, Santa Cruz de Tenerife, Spain; 10 Pulmonary Medicine Department, Hospital Universitario de Canarias, 38320 San Cristóbal de La Laguna, Santa Cruz de Tenerife, Spain; hemilykaterine@hotmail.com; 11 Unit Service of Microbiology, Hospital Universitario Nuestra Señora de La Candelaria, 38010 Santa Cruz de Tenerife, Spain; juliaalcoba@yahoo.com; 12 Department of Immunology, IIS-Fundación Jiménez Díaz, 28040 Madrid, Spain; jose.canas@fjd.es (J.A.C.); jose.rodrigom@quironsalud.es (J.M.R.M.); vpozo@fjd.es (V.d.P.); 13 CIBER de Enfermedades Respiratorias, Instituto de Salud Carlos III, 28029 Madrid, Spain; linaiperezmendez@gmail.com (L.I.P.M.); jesus.villar54@gmail.com (J.V.); 14 Division of Clinical Epidemiology and Biostatistics, Research Unit Hospital Universitario de N.S de Candelaria and Primary Care Management, 38010 Santa Cruz de Tenerife, Spain; 15 Department of Biochemistry, Microbiology, Cell Biology and Genetics, Universidad de La Laguna, 38200 San Cristóbal de La Laguna, Santa Cruz de Tenerife, Spain; mnhdez@ull.edu.es; 16 Instituto Universitario de Enfermedades Tropicales y Salud Pública de Canarias (IUETSPC), Universidad de La Laguna, 38200 San Cristóbal de La Laguna, Santa Cruz de Tenerife, Spain; 17 Multidisciplinary Organ Dysfunction Evaluation Research Network (MODERN), Research Unit, Hospital Universitario Dr. Negrín, 35019 Las Palmas, Spain; 18 Instituto de Tecnologías Biomédicas (ITB), Universidad de La Laguna, 38200 San Cristóbal de La Laguna, Santa Cruz de Tenerife, Spain

**Keywords:** asthma, exacerbation, microbiome, genetics, precision medicine, respiratory disease, biomarker

## Abstract

Asthma exacerbations are a major contributor to the global disease burden, but no significant predictive biomarkers are known. The Genomics and Metagenomics of Asthma Severity (GEMAS) study aims to assess the role of genomics and the microbiome in severe asthma exacerbations. Here, we present the design of GEMAS and the characteristics of patients recruited from March 2018 to March 2020. Different biological samples and demographic and clinical variables were collected from asthma patients recruited by allergy and pulmonary medicine units in several hospitals from Spain. Cases and controls were defined by the presence/absence of severe asthma exacerbations in the past year (oral corticosteroid use, emergency room visits, and/or asthma-related hospitalizations). A total of 137 cases and 120 controls were recruited. After stratifying by recruitment location (i.e., Canary Islands and Basque Country), cases and controls did not differ for most demographic and clinical variables (*p* > 0.05). However, cases showed a higher proportion of characteristics inherent to asthma exacerbations (impaired lung function, severe disease, uncontrolled asthma, gastroesophageal reflux, and use of asthma medications) compared to controls (*p* < 0.05). Similar results were found after stratification by recruitment unit. Thereby, asthma patients enrolled in GEMAS are balanced for potential confounders and have clinical characteristics that support the phenotype definition. GEMAS will improve the knowledge of potential biomarkers of asthma exacerbations.

## 1. Introduction

Asthma is one of the most prevalent chronic respiratory diseases worldwide. Up to 330 million individuals have asthma and 346,000 deaths per year are related to this disease [1]. In Spain, the prevalence of asthma in children is about 10% [2], although there is a high variation among regions, reaching the highest value (17%) in the Canary Islands [3,4]. Asthma is defined as a heterogeneous respiratory disease characterized by chronic airway inflammation, variable expiratory airflow limitation, and airway hyperresponsiveness to different triggers [5]. Characteristic symptoms of asthma, including wheeze, dyspnea, chest tightness, and cough, may resolve spontaneously or may be controlled with adequate pharmacological therapies [2,5]. Nonetheless, patients with asthma can experience worsening episodes of the disease known as exacerbations, which carry a considerable burden to patients and health systems [5]. Asthma exacerbations limit the daily routine of patients, and they are related to hospitalizations, disability, and higher mortality [1,5]. Moreover, they are associated with high healthcare expenses derived from emergency room (ER) visits, hospitalizations, increased use of medication, and lost workdays [6]. Despite its clinical and economical relevance, no biomarkers are available to predict asthma exacerbations and the only good predictor is the history of at least one episode of an asthma exacerbation in the past year [7]. Therefore, the development of new strategies and biomarkers to identify patients at high risk of asthma exacerbations is needed [8].

Asthma is a multifactorial disease in which genetic variation has a key role. The heritability of asthma has been estimated at 65% in adults and even higher in childhood asthma [9]. As in most polygenic and complex diseases, single nucleotide polymorphisms (SNPs) are the most widely studied genetic variants. They consist of a variation in only one nucleotide with a population frequency higher than 1%. Hundreds of thousands of SNPs can be genotyped by micro-arrays and because of linkage disequilibrium patterns, millions of SNPs can be inferred using imputation reference panels. Therefore, genome-wide association studies (GWAS) allow for assessing genetic variation throughout the whole genome without any prior hypothesis of the processes involved in, unlike candidate-gene studies that focus on targeted genes with a previous relationship with the disease [10]. While the genetic variation of asthma and other related phenotypes, such as treatment response, have been explored by GWAS, the genetic basis of severe asthma exacerbations has been scarcely investigated through candidate-gene approaches analyzing genes involved in asthma susceptibility [9,11,12,13].

Similar to human genetic variation, the human microbiota has a role in the development of the immune system and allergic diseases such as asthma [14]. Advances in next-generation sequencing (NGS) techniques have allowed a more accurate microbial characterization based on its genetic make-up (known as the microbiome) regardless of whether the microorganisms are culturable or not. Targeted sequencing of the 16S ribosomal RNA (16S rRNA) gene, a specific bacterial taxonomic marker, has been extensively used to infer the bacterial abundance and diversity of the microbiota [15]. This approach has been applied to describe the microbial diversity in the airways and the continuity in bacterial communities through the oral cavity, upper airways, and lungs [16,17]. In relation to asthma, the alteration (dysbiosis) of the nasopharyngeal or the salivary microbiome is associated with asthma susceptibility and different phenotypes of severe asthma [18,19,20]. Moreover, respiratory pathogens constitute known triggers for asthma exacerbations and are involved in the development of airway inflammation [16,21].

Despite all this evidence, the role of the salivary and nasopharyngeal microbiome in the development of asthma exacerbations has been scarcely investigated [22,23,24]. Furthermore, regardless of the interplay between host genetics and the microbiome [25], the integrated effect of both layers in the development of asthma exacerbations is still unknown. The integration of clinical data, human genetic variants, and the microbiome has been proposed as a novel approach moving forward the asthma pathogenesis knowledge and precision medicine in asthma management [8,26]. Moreover, well-conducted observational studies are needed to identify potential pharmacogenetic biomarkers before assessing their applicability in precision medicine through clinical trials [27]. In this context, the Genomics and Metagenomics of Asthma Severity (GEMAS) study has emerged to assess the role of genomics, the microbiome, and the interaction between them in the development of asthma exacerbations in Spanish patients with asthma. This manuscript aims to present the rationale, study design, and the demographic and clinical characteristics of the patients recruited in GEMAS between March 2018 and March 2020.

## 2. Materials and Methods

### 2.1. Study Design and Data Collection

The GEMAS study is an ongoing retrospective multicenter study coordinated by the Genomics and Health group from Universidad de La Laguna, Tenerife, Spain. Patients are recruited from the allergy and/or pulmonary medicine units from three hospitals located at the Canary Islands (Hospital Universitario de Canarias, Hospital Universitario Nuestra Señora de Candelaria, and Hospital General de La Palma), and in the division of pediatric respiratory medicine of the Hospital Universitario Donostia in the Basque Country. The study was done according to the code of ethics of the World Medical Association (Declaration of Helsinki) and approval was obtained from the ethics committees of participant centers (approval 29/17 for the Canary Islands hospitals and PI2019077 for Hospital Universitario Donostia). This study is registered in the ClinicalTrials.gov database of the National Institutes of Health (NIH) (identifier: NCT04501926).

Patients were eligible according to the following inclusion criteria: (1) male or female aged ≥8 and ≤85 years, (2) physician diagnosis of asthma according to the Global Initiative for Asthma (GINA) guidelines [5], and (3) treated on GINA step 1–5. Patients were excluded according to the following exclusion criteria: (1) one or more grandparents of non-European origin, (2) pregnancy, (3) coexistence of other chronic pulmonary disorders including cystic fibrosis, chronic obstructive pulmonary disease (emphysema or chronic bronchitis), or congenital disorders of the lungs or airways, and (4) known family relatedness (first or second degree) with another participant already included in the study. Asthma patients from allergy and/or pulmonary medicine units were ≥14 years old and those recruited in the division of pediatric respiratory medicine were aged between 8 and 16 years. Patients under 18 years old provided their assent (with signature for those aged 12–17 years) and one of their legal guardians signed the informed consent. Participants older than 18 years old provided their informed consent to participate in the study. Clinical and demographic variables were registered using a standardized questionnaire. Briefly, these variables include gender, age, height, weight, smoking exposure, educational level, home environment, household pets, lung function measurements, fraction of exhaled nitric oxide (FeNO), total serum immunoglobulin E (IgE) and blood eosinophil levels, comorbidities (otorhinolaryngology diseases, gastroesophageal reflux [GERD], and sleep apnea), atopy tests, the presence of other allergic phenotypes, family history of allergic diseases, age of asthma onset, asthma severity, asthma control, asthma exacerbations, and pharmacological treatment.

The main outcome assessed in this study was severe asthma exacerbations, defined by one of the following events related to asthma in the past year: (1) emergency room (ER) visits, (2) hospitalizations, and/or (3) oral corticosteroids (OCS) use. Case and control status were defined as the presence or absence of asthma exacerbations in the year before enrollment, respectively. Asthma exacerbations, as well as the use of asthma medication were recorded for three time points prior to recruitment: the past week, the past six months, and the past 12 months. Medication adherence was evaluated by the Medication Adherence Report Scale (MARS-5). Asthma control was assessed by the asthma control questionnaire (ACQ) score and the severity of the disease in each patient was classified based on treatment step according to GINA 2020 [5]. In addition, the following variables that may influence the microbiome composition were recorded: (1) the use of antibiotics in the past two months, (2) health condition during the sampling (presence of cavities, gingivitis, mouth sores, cold, or flu, and pregnancy and lactation status when appropriate), (3) sampling conditions within 30 min and 2 h prior sampling (inhaled corticosteroids use, any non-alcoholic or alcoholic drinking, eating, teeth brushing, smoking, and chewing gum), and (4) vaccination in the past month (i.e., influenza and pneumococcal bacteria).

Due to the multicenter nature of the study, quality control of the registered data was performed in order to ensure consistent results across recruiter centers. Physicians were instructed to select eligible patients to recruit and to collect the biological samples following common procedures, and standardized questionnaires were completed minimizing missing data. Additionally, the data recorded in the questionnaires were registered and double-checked in a unique database by at least two independent researchers. Implausible data entries or inconsistencies between variables detected in the questionnaires were corrected by checking the medical records.

### 2.2. Clinical Assessment

Total serum IgE and blood eosinophil levels were assessed by blood tests. Atopy status was evaluated by skin prick tests and/or specific IgE levels. These tests assessed the most common aeroallergens of each recruiting center, including at least: mites (*Dermatophagoides pteronyssinus*, *Dermatophagoides farinae*, and *Blomia tropicalis*), fungi (*Alternaria alternata*), dog and cat epithelium, grass mix (*Anthoxanthum odoratum*, *Cynodon dactylon*, *Dactylis glomerata*, *Festuca pratensis*, *Holcus lanatus*, *Lolium perenne*, *Phleum pratense*, *Poa pratensis*, and/or *Secale cereale*), mugwort (*Artemisia vulgaris*), and *Parietaria*. The skin prick test included both positive (histamine) and negative controls. Atopy was defined by at least one positive result from any of the allergens evaluated, based on a wheal diameter ≥3 mm larger than the negative control in the skin prick test or specific IgE levels >0.35 UI/mL.

Lung function was assessed by baseline and post-bronchodilator measurements of the forced expiratory volume in the first second (FEV_1_), the forced vital capacity (FVC), and the FEV_1_/FVC ratio. Spirometry and the bronchodilator test were performed according to the American Thoracic Society and the European Respiratory Society (ATS/ERS) international guidelines [28]. Subjects were instructed to withhold short-acting bronchodilators for 8 h and long-acting bronchodilators for 24 h prior to pulmonary function testing. Patients must avoid smoking or vaping in the previous hour, do not consume intoxicants in the eight hours before the test (including alcohol), do not perform vigorous exercise in the previous hour, and do not wear clothes that may restrict the lung function. Moreover, based on recommendations from the Spanish Society of Pulmonology and Thoracic Surgery (SEPAR) [29], patients were also recommended to avoid the consumption of stimulant drinks (including coffee or tea) and large meals in the previous 2–3 h. Predicted values and z-scores of lung function measurements were estimated using the Global Lung Function Initiative (GLI) 2012 equations [30]. Bronchodilator response (BDR) was calculated as (post-FEV_1_ − preFEV_1_)/preFEV_1_. FeNO levels were assessed at Hospital Universitario Donostia following ATS/ERS international guidelines [31].

Body mass index (BMI) was calculated based on weight and height, and obesity was defined following the World Health Organization (WHO) criteria as BMI ≥ 30 kg/m^2^ for participants older than 18 years. For those aged ≤18 years, BMI-for-age z-scores were calculated based on the WHO growth reference for children aged between 5–19 years, and obesity was defined as a value greater than two standard deviations above the reference median [32].

### 2.3. Biological Sample Collection and Storage

From each patient, saliva, nasal, and pharyngeal samples were obtained for bacterial deoxyribonucleic acid (DNA) extraction. One milliliter of saliva was collected in Oragene OMNIgene^®^ ORAL OM501 tubes (DNA Genotek, Inc., Ottawa, ON, Canada) to ensure the stability and integrity of bacterial communities. Following standardized procedures, nasal and pharyngeal samples were obtained with sterile swabs stored in AMIES transport medium (DeltaSwab Amies, Deltalab, Barcelona, Spain). Moreover, 4 mL of blood were drawn in tubes with ethylenediaminetetraacetic acid (EDTA) for human DNA extraction and 2.5 mL were collected with PAXgene^®^ Blood RNA Tubes (PreAnalytiX, Feldbachstrasse, Switzerland) to stabilize the intracellular human ribonucleic acid (RNA). EDTA tubes were drawn first to avoid changes in gene expression due to the phlebotomy procedure. Samples and data from patients from the Basque country included in this study were provided by the Basque Biobank/Biodonostia Node and were processed following standard operating procedures with the appropriate approval of the Ethical and Scientific Committees. Samples were stored at −20 °C at each recruiter center, and they were transported and stored at the same temperature at Universidad de La Laguna until processing, except for the PAXgene tubes that were stored at −80 °C.

### 2.4. Processing of Samples for Future Genomic and Metagenomic Studies

Bacterial DNA extraction was performed from saliva, nasal, and pharyngeal samples following a protocol that includes a mechanical pre-lysis of bacterial cells with the Pathogen Lysis Tubes S kit (Qiagen, Hilden, Germany) and the isolation and purification of nucleic acids with the spin column-based kit QIAamp UCP Pathogen Mini kit (Qiagen). Human DNA extraction from blood samples was performed using the Illustra blood genomicPrep Mini Spin Kit (GE Healthcare, Amersham, UK) following the manufacturer’s instructions. RNA extraction was performed with the NucleoSpin^®^ RNA Blood kit (MACHEREY-NAGEL, Düren, Germany). DNA and RNA samples are stored at −20 °C and −80 °C, respectively, until they are analyzed.

The human microbiome will be assessed by targeted sequencing of the V3–V4 region of the 16S ribosomal RNA (16S rRNA) gene. This region has been selected because it provides information with high coverage of the bacterial microbiome at genera-level and is the most used region in human microbiome studies [33]. Paired-end sequencing (300 nucleotides × 2) will be performed using the NGS MiSeq platform and the MiSeq Reagent Kit v3 (Illumina, Inc., San Diego, USA). On the other hand, human DNA samples will be genotyped using the Infinium Global Screening Array-24 kit v3.0 (Illumina, Inc.) to interrogate up to 642,824 single nucleotide polymorphisms (SNPs) across the whole genome.

### 2.5. Statistical Analysis for the Comparison of Demographic and Clinical Characteristics between Cases and Controls

A comparison of the demographic and clinical characteristics of cases and controls was performed separately for the participants recruited in the Canary Islands and the Basque country. Additionally, a stratified analysis was performed for each of the recruitment units (i.e., allergy or pulmonary medicine). Continuous variables with a normal distribution were summarized using mean values and standard deviation, and those non-normally distributed by the median values and the interquartile range. Differences between groups were evaluated using the t-test and the Mann–Whitney U-test for continuous variables with normal and non-normal distribution, respectively. Categorical variables were summarized by counts in each category and percentages, and they were compared between cases and controls using the Fisher’s exact test. Statistical significance was declared at *p*-value < 0.05.

## 3. Results

A total of 257 individuals with asthma (137 cases and 120 controls) have been recruited (Table 1, Box 1). Saliva, nasal, pharyngeal, and blood samples are available from all subjects, and blood samples in PAXgene tubes have been collected from a subset of 39 adults with asthma from the Canary Islands (21 cases and 18 controls).

The comparison of cases (n = 103) and controls (n = 102) recruited in the Canary Islands showed no differences between both groups for age, gender, tobacco smoking exposure, educational level, home environment, or household pets (*p*-value > 0.05, Table 1). Moreover, some clinical variables related to asthma, such as the presence of atopy and other allergic phenotypes, total IgE and eosinophil levels, family history of asthma and allergic diseases, age of asthma onset, and medication adherence were similar between both groups (*p*-value > 0.05).

Cases had a higher lung function impairment compared to controls (median predicted pre-FEV_1_: 83.4% vs. 90.3%, *p*-value = 0.003; median predicted pre-FVC: 87.7% vs. 94.2%, *p*-value = 0.020). When asthma-related comorbidities were assessed, only GERD was more frequent in patients who had exacerbations in the past year compared to those who did not have exacerbations (32.7% vs. 15.7%, *p*-value = 0.005). Additionally, severe asthma and poorly controlled asthma were more frequent in cases compared to controls (*p*-value = 0.005 and *p*-value = 1.2 × 10^−5^, respectively). Moreover, patients with asthma exacerbations required higher administration of both reliever and controller asthma medications in the past year, such as short-acting beta agonists (SABA), inhaled corticosteroids (ICS), long-acting beta agonists (LABA), or OCS (*p*-value < 0.05, Table 1), and were more frequently treated with antibiotics in the past two months compared to controls (44.1% vs. 17.8%, *p*-value = 6.8 × 10^−5^). Similar trends of lung function impairment, coexistence of GERD, presence of severe and poorly controlled asthma, and use of asthma medications were observed when cases and controls were stratified based on recruitment unit (allergy and pulmonary medicine units) (Table S1).

Box 1 **Summary of the main characteristics of patients enrolled in the Genomics and Metagenomics of Asthma Severity (GEMAS) study.**

- A total of 257 individuals with asthma have been recruited between March 2018 and March 2020 (137 cases and 120 controls).
- Cases and controls recruited in the Canary Islands and in the Basque Country did not differ for many potential confounders for the future genomic and microbiome analyses (e.g., age, gender, tobacco exposure, comorbidities, etc.).
- Cases from the Canary Islands have clinical characteristics that support the definition of asthma exacerbations (i.e., impaired lung function, severe and uncontrolled asthma, coexistence of GERD, and use of asthma medication).
- Children from the Basque Country who had asthma exacerbations in the past year have higher proportion of severe asthma and OCS use, and worse medication adherence than controls.

From the 52 children with asthma recruited in the Basque country, 34 were classified as cases and 18 as controls (Table 1). There were no differences between both groups in any of the clinical and demographic variables except for severe asthma (94.1% vs. 22.2%, *p*-value = 1.7 × 10^−7^) and treatment with OCS (94.1% vs. 5.6%, *p*-value = 1.3 × 10^−10^), which were more frequent in exacerbators. Additionally, patients with exacerbations had worse self-reported medication adherence than controls (*p*-value = 0.033, Table 1). No differences either in lung function or FeNO were found between children who developed asthma exacerbations and those who did not (*p*-value > 0.05, Table 1).

## 4. Discussion

GEMAS is a study aimed at investigating the role of genomics and the human microbiome in asthma exacerbations that has included, so far, a total of 257 children and adults with asthma from the Canary Islands and the Basque Country. Patients were divided into cases and controls according to the presence/absence of asthma exacerbations, obtaining a well-balanced distribution of individuals in the two groups. There were no differences between groups in many potential confounders for the genomic and microbiome analyses and the observed dissimilarities support the definition of cases and controls since they are based on characteristics inherent to the trait [34]. From the patients recruited in the Canary Islands, those who developed asthma exacerbations showed lower lung function and had a higher proportion of poor asthma control, more severe disease, the coexistence of GERD, and the use of reliever and controller medications than controls. Moreover, the group of cases showed higher rates of prescription of antibiotics in the last two months, which was expected given the relationship between respiratory infections and asthma exacerbations [21]. From children recruited in the Basque Country, cases and controls were also balanced for potential confounders except for the use of OCS and self-reported medication adherence. Unlike adult patients, lung function in children did not show greater deterioration in cases with asthma exacerbations than in controls, which was expected since the impairment of lung function because of asthma is a chronic process that worsens over time and with the frequency of exacerbations [35].

Across the literature, similarities and differences between adulthood and childhood asthma have been described [36,37]. Indeed, differences in some clinical variables from the Canary Islands and Basque Country patients might be related to the proportion of asthma phenotypes in these two groups. All patients from the Basque Country were children (aged between 8 and 16 years) and have a median age of asthma onset of 2.3 years and a high frequency of clinical variables related to allergy. In addition, 80% of them have atopy, 83% other allergic phenotypes (e.g., rhinitis and/or dermatitis), and 81% family history of other allergic diseases. All these features are characteristic of an early-onset allergic T_H_2 asthma [5,37], so it is expected that a higher incidence of this phenotype will occur in this group. Besides, T_H_2 inflammation has been also strongly associated with type I hypersensitivity allergy (which is mediated by IgE) and eosinophilic inflammation [5,37], which can explain the higher levels of total serum IgE (median 462.0 vs. 137.9 UI/mL, *p*-value = 0.013) and blood eosinophils (median 500.0 vs. 300.0 cells/mm^3^, *p*-value = 0.002) in this population when compared with the Canary Islands patients. In contrast, patients recruited in the Canary Islands include mostly adult asthma patients. Therefore, it is expected that not only childhood-onset but also adult-onset asthma phenotypes (e.g., neutrophilic, obesity-related, smoking-associated…) coexist in this group. Indeed, the median age of asthma onset in the Canary Islands patients was 18 years (interquartile range: 6.0–40.0 years). As age of asthma onset increases, it is expected a higher incidence of non-T_H_2 asthma and thereby, other mechanisms different than IgE-mediated response or eosinophilic inflammation may underly adult-onset asthma [37].

Regarding the genetics of asthma, adulthood and childhood asthma phenotypes have a similar but not identical genetic basis [38]. Moreover, although several genetic variants associated with asthma-related traits, such as treatment response, have demonstrated similar effects both in adults and children, many of them are not shared and aging has a mitigating effect [12]. Nevertheless, the overlap between polymorphisms related to asthma exacerbations in adults and children has been scarcely investigated [11]. Besides, it is well known that age is closely related to the composition of the human microbiome [39], which can limit the comparison between adult and children studies. Indeed, microbiome studies of asthma exacerbations conducted in children have shown consistent findings, such as the presence of *Moraxella* in the nasal microbiome as a risk factor of exacerbations [22,24], but these findings differ from those identified in adults [23]. Given the limited number of studies, definitive conclusions cannot be established, and GEMAS can provide independent insights into the comparison between pediatric and adult populations.

The GEMAS study has several strengths and limitations that must be considered. Regarding the strengths, to the best of our knowledge, GEMAS is the first study of asthma exacerbations where different non-invasive samples (saliva, nasal, and pharyngeal swabs) have been collected. Moreover, the use of standardized and common procedures makes GEMAS an open-collaboration study to participate in meta-analyses to disentangle the role of genomics not only in asthma exacerbations but also in other allergic and respiratory traits. Indeed, the characteristics of GEMAS will allow collaboration with other studies of Spanish asthma patients recruited in different regions of the country, such as the MEGA project [40], to improve the knowledge of asthma in this population. Finally, the fact that genotype and microbiome data will be available in GEMAS from both children and adults with asthma will allow investigation into the interaction of these two layers in two differentiated asthma phenotypes.

On the other hand, some limitations need to be acknowledged. First, despite assessing potential variables that may disturb the microbiome, many other potential confounders of microbiome assessment may be uncontrolled (e.g., use of probiotics, gastrointestinal diseases, evidence of oral candidiasis, etc.) [41]. Moreover, the use of antibiotics was quite extended among patients who developed asthma exacerbations and their effects on the human microbiome have a high interindividual variation [42]. However, the fact that antibiotic use is recorded will allow us to take this variable into account in the statistical analyses. Second, the multicenter nature study of the microbiome may limit the power to detect differences in the human microbiome composition, since it is highly conditioned by the environment [43]. Third, the current sample is limited, and healthy individuals have not been recruited as additional controls. However, this limitation will be overcome in future stages of the study, since patient enrollment is still ongoing.

## 5. Conclusions

GEMAS is a promising study with well-balanced groups of asthma patients with and without asthma exacerbations that will contribute to improving the knowledge of the scarcely investigated role of genomics and the human microbiome in the development of asthma exacerbations. The clinical characteristics of the enrolled patients support the definitions of cases and controls, and the use of common and standardized procedures will allow for the collaboration with other studies. Moreover, the collection of non-invasive samples to investigate the human microbiome will help to establish potential biomarkers with better clinical applicability that could be included in further clinical studies to move towards precision medicine.

## Figures and Tables

**Table 1:** Description of patients with asthma recruited in the GEMAS study.

	Canary Islands Patients				Basque Country Patients			
	Sample Size (n)	Controls (n = 102)	Cases (n = 103)	*p*-Value	Sample Size (n)	Controls (n = 18)	Cases (n = 34)	*p*-Value
Age (years)	205	50.5 (33.0–62.0)	46.0 (29.5–59.5)	0.253	52	10.0 (9.0–11.8)	11.0 (9.0–12.8)	0.507
Gender (female)	205	65 (63.7)	71 (68.9)	0.462	52	4 (22.2)	16 (47.1)	0.133
Ever smoker or secondhand smoke exposure ^a^	202	29 (28.7)	38 (37.6)	0.232	52	9 (50.0)	19 (55.9)	0.774
Lung function								
pre-FEV_1_ (% predicted)	197	90.3 (76.1–101.9)	83.4 (69.2–92.6)	**0.003**	48	95.8 (89.2–102.3)	99.4 (89.4–106.4)	0.455
pre-FEV_1_ (z-score)	197	−0.8 (1.3)	−1.3 (1.3)	**0.006**	48	−0.3 (0.8)	−0.1 (1.2)	0.415
pre-FVC (% predicted)	196	94.2 (81.3–102.6)	87.7 (79.3–96.5)	**0.020**	48	102.2 (92.9–108.3)	104.4 (92.1–109.9)	0.579
pre-FVC (z-score)	196	−0.5 (1.1)	−0.9 (1.1)	**0.012**	48	0.0 (0.8)	0.2 (1.0)	0.511
pre-FEV_1_/FVC (%)	196	79.1 (72.4–83.5)	75.9 (68.3–83.2)	0.138	48	83.9 (79.2–86.9)	83.8 (80.4–89.2)	0.429
BDR (%)	71	4.7 (1.8–10.5)	9.0 (3.1–15.0)	0.166	48	3.4 (1.8–5.3)	2.4 (−0.5–3.6)	0.344
FeNO (ppb)	NA	NA	NA	NA	48	15.0 (9.0–39.7)	14.9 (8.6–28.1)	0.491
Total IgE levels (UI/mL)	187	149.8 (42.1–434.5)	125.7 (39.3–449.3)	0.641	17	902.5 (287.5–2402.5)	450.0 (172.5–635.5)	0.216
Absolute eosinophil count (cells/µL)	192	300.0 (200.0–500.0)	300.0 (100.0–500.0)	0.209	15	750.0 (467.5–1027.5)	500.0 (425.0–710.0)	0.412
Eosinophil percentage (%)	186	4.3 (2.6–6.8)	3.7 (1.5–7.0)	0.190	10	5.1 (5.0–11.3)	6.0 (3.8–8.7)	0.933
Comorbidities					52			
Otorhinolaryngology disease	202	24 (23.8)	29 (28.7)	0.523	51	2 (11.8)	5 (14.7)	1.000
Gastroesophageal reflux	203	16 (15.7)	33 (32.7)	**0.005**	52	1 (5.6)	0 (0)	0.346
Sleep apnea	202	15 (15.0)	12 (11.8)	0.540	52	1 (5.6)	1 (2.9)	1.000
Obesity	197	37 (36.6)	33 (34.4)	0.768	49	3 (17.6)	4 (12.5)	0.681
Atopy	179	69 (76.7)	70 (78.7)	0.858	50	14 (82.4)	26 (78.8)	1.000
Other allergic phenotypes	202	72 (70.6)	74 (74.0)	0.639	52	15 (83.3)	28 (82.4)	1.000
Rhinitis	202	65 (63.7)	67 (67.0)	0.659	52	10 (55.6)	23 (67.6)	0.546
Dermatitis	202	18 (17.6)	20 (20.0)	0.721	52	6 (33.3)	9 (26.5)	0.749
Drug allergy	202	17 (16.7)	22 (22.0)	0.376	52	1 (5.6)	0 (0)	0.346
Food allergy	202	7 (6.9)	12 (12.0)	0.236	52	6 (33.3)	5 (14.7)	0.159
Age of asthma onset (years)	188	16.0 (5.0–40.0)	20.0 (6.0–44.8)	0.572	48	2.5 (1.8–4.8)	2.3 (1.0–4.0)	0.423
Family history								
Allergy	205	70 (68.6)	71 (68.9)	1.000	52	15 (83.3)	27 (79.4)	1.000
Asthma	205	44 (43.1)	46 (44.7)	0.888	52	11 (61.1)	18 (52.9)	0.770
Asthma exacerbations	205				52			
OCS use	205	0 (0)	70 (68.0)	NA	52	0 (0)	34 (100)	NA
ER visits	205	0 (0)	93 (90.3)	NA	52	0 (0)	20 (58.8)	NA
Hospitalizations	205	0 (0)	20 (19.4)	NA	52	0 (0)	5 (14.7)	NA
Asthma severity	200				52			
Mild		8 (7.9)	0 (0)	**0.007**		3 (16.7)	1 (2.9)	0.113
Moderate		9 (8.9)	4 (4.0)	**0.251**		11 (61.1)	1 (2.9)	**5.3 × 10^−6^**
Severe		84 (83.2)	95 (96)	**0.005**		4 (22.2)	32 (94.1)	**1.7 × 10^−7^**
Asthma control	191				48			
Well controlled		55 (56.1)	26 (28.0)	**1.3 × 10^−4^**		14 (82.4)	26 (83.9)	1.000
Partially controlled		29 (29.6)	27 (29.0)	1.000		3 (17.6)	2 (6.5)	0.331
Poorly controlled		14 (14.3)	40 (43.0)	**1.2 × 10^−5^**		0 (0)	3 (9.7)	0.543
Pharmacological treatment								
SABA	205	47 (46.1)	79 (77.5)	**6.5 × 10^−6^**	52	13 (72.2)	31 (91.2)	0.108
ICS	205	95 (93.1)	103 (100)	**0.007**	52	18 (100)	34 (100)	NA
LABA	205	90 (88.2)	101 (98.1)	**0.006**	52	11 (61.1)	26 (76.5)	0.337
LTRA	205	50 (49.0)	63 (61.8)	0.091	51	0 (0)	1 (3.0)	1.000
OCS	204	7 (6.9)	51 (50.5)	**1.3 × 10^−12^**	52	1 (5.6)	32 (94.1)	**1.3 × 10^−10^**
SAMA	205	19 (18.6)	42 (41.2)	**7.0 × 10^−4^**	52	0 (0)	0 (0)	NA
LAMA	205	25 (24.5)	31 (30.4)	0.433	51	0 (0)	0 (0)	NA
Theophylline	205	0 (0)	9 (8.8)	**0.003**	52	0 (0)	0 (0)	NA
Antihistamines	205	58 (56.9)	74 (72.5)	**0.028**	52	3 (16.7)	14 (41.2)	0.120
Azithromycin	205	5 (4.9)	15 (14.7)	**0.032**	52	1 (5.6)	10 (29.4)	0.073
Biological therapies	205	14 (13.7)	16 (15.7)	0.844	52	0 (0)	1 (2.9)	1.000
Immunotherapy	204	21 (20.6)	17 (16.8)	0.590	51	0 (0)	0 (0)	NA
Antibiotics	205	18 (17.8)	45 (44.1)	**6.8 × 10^−5^**	52	1 (5.6)	6 (17.6)	0.399
Medication adherence	203	25.0 (23.0–25.0)	25.0 (23.0–25.0)	0.121	52	24.0 (24.0–25.0)	24.0 (22.3–25.0)	**0.033**
Home environment (rural)	204	52 (51.0)	60 (58.8)	0.325	52	6 (33.3)	4 (11.8)	0.076
Household pets	201	48 (48.5)	49 (48.0)	1.000	52	1 (5.6)	9 (26.5)	0.136
Education level ^a^	204				51			
No schooling completed		2 (2.0)	1 (1.0)	1.000		0 (0)	0 (0)	NA
Lower secondary education		40 (39.2)	39 (38.2)	1.000		3 (17.6)	7 (20.6)	1.000
Higher secondary education		37 (36.3)	41 (40.2)	0.666		8 (47.1)	9 (26.5)	0.208
Higher education		23 (22.5)	21 (20.6)	0.865		6 (35.3)	18 (52.9)	0.372

^a^ Data referred to the parents in case of children from the Basque Country. Descriptives are represented by the mean (standard deviation) and the median (interquartile range) values for continuous variables with a normal and non-normal distribution, respectively, and the count (proportion) for categorical variables. Differences between groups were evaluated using the *t*-test and the Mann–Whitney U-test for continuous variables with normal and non-normal distribution, respectively, and Fisher’s exact test for categorical variables. Statistically significant differences are indicated in boldface (*p* < 0.05). **Abbreviations:** FEV_1_: Forced expiratory volume in the first second; FVC: Forced vital capacity; BDR: Bronchodilator response; FeNO: Fraction of exhaled nitric oxide; IgE: Immunoglobulin E; OCS: Oral corticosteroids; ER: Emergency room; SABA: Short-acting beta agonists; ICS: Inhaled corticosteroids; LABA: Long-acting beta agonists; LTRA: Leukotriene receptor antagonists; SAMA: Short-acting muscarinic antagonists; LAMA: Long-acting muscarinic antagonists; NA: Not applicable.

## Supplementary Materials

The following are available online at https://www.mdpi.com/2075-4426/10/3/123/s1, Table S1: Description of asthma patients recruited in the Canary Islands.

## Abbreviations

16S rRNA	16S ribosomal ribonucleic acid
ACQ	Asthma Control Questionnaire
ATS	American Thoracic Society
BDR	Bronchodilator response
BMI	Body mass index
DNA	Deoxyribonucleic acid
EDTA	Ethylenediaminetetraacetic acid
ER	Emergency room
ERS	European Respiratory Society
FeNO	Fraction of exhaled nitric oxide
FEV_1_	Forced expiratory volume in the first second
FVC	Forced vital capacity
GEMAS	Genomics and Metagenomics of Asthma Severity
GERD	Gastroesophageal reflux disease
GINA	Global Initiative for Asthma
GLI	Global Lung Function Initiative
GWAS	Genome-wide association study
ICS	Inhaled corticosteroids
IgE	Immunoglobulin E
LABA	Long-acting beta agonists
LTRA	Leukotriene receptor antagonists
MARS-5	Medication Adherence Report Scale
MEGA	Mechanisms Involved in the Genesis and Disease Course of Asthma
NIH	National Institutes of Health
NGS	Next-generation sequencing
OCS	Oral corticosteroids
RNA	Ribonucleic acid
SABA	Short-acting beta agonists
SAMA	Short-acting muscarinic antagonists
SEPAR	Sociedad Española de Neumología y Cirugía Torácica
SNP	Single nucleotide polymorphism
WHO	World Health Organization
