# Supplementary material for: The Genomics and Metagenomics of Asthma Severity (GEMAS) Study: Rationale and Design
Source: J Pers Med. 2020 Sep 11;10(3):123. doi: 10.3390/jpm10030123 (PMC7563269; doi:10.3390/jpm10030123)
Supplement: Supplementary file 1 [file jpm-10-00123-s001.pdf]

Supplementary Material. The Genomics and Metagenomics of Asthma Severity (GEMAS) study: rationale and design

Table S1. Description of patients with asthma recruited in the Canary Islands.

		All patients			Allergy units			Pulmonary medicine units		
	Sample size (n)	Controls (n=102)	Cases (n=103)	*p*-value	Controls (n=31)	Cases (n=43)	*p*-value	Controls (n=71)	Cases (n=60)	*p*-value
Age (years)	205	50.5 (33.0-62.0)	46.0 (29.5-59.5)	0.253	33.0 (21.5-45.0)	33.0 (20.5-49.0)	0.832	55.0 (44.0-66.0)	51.5 (37.8-64.5)	0.492
Gender (female)	205	65 (63.7)	71 (68.9)	0.462	20 (64.5)	29 (67.4)	0.808	45 (63.4)	42 (70.0)	0.462
Ever smoker	202	29 (28.7)	38 (37.6)	0.232	4 (13.3)	13 (31.7)	0.095	25 (35.2)	25 (41.7)	0.475
Lung function										
pre-FEV_1 (% predicted)	197	90.3 (76.1-101.9)	83.4 (69.2-92.6)	**0.003**	88.5 (72.9-95.2)	79.4 (68.4-90.7)	0.153	92.3 (79.6-102.3)	85.7 (71.3-93.2)	**0.017**
pre-FEV_1 (z-score)	197	-0.8 (1.3)	-1.3 (1.3)	**0.006**	-1.2 (1.5)	-1.5 (1.4)	0.316	-0.7 (1.1)	-1.2 (1.2)	**0.014**
pre-FVC (% predicted)	196	94.2 (81.3-102.6)	87.7 (79.3-96.5)	**0.020**	95.2 (82.3-101.0)	89.4 (79.9-96.1)	0.159	93.6 (80.8-103.6)	86.3 (78.7-97.0)	0.052
pre-FVC (Z-score)	196	-0.5 (1.1)	-0.9 (1.1)	**0.012**	-0.5 (1.0)	-0.9 (1.1)	0.125	-0.6 (1.1)	-0.9 (1.0)	**0.045**
pre-FEV_1/FVC (%)	196	79.1 (72.4-83.5)	75.9 (68.3-83.2)	0.138	78.1 (70.0-81.8)	73.4 (67.8-83.2)	0.590	79.5 (72.5-83.6)	76.8 (71.1-83.2)	0.238
BDR (%)	72	4.7 (1.8-10.5)	9.0 (3.1-15.0)	0.166	5.7 (2.8-13.4)	10.8 (3.7-17.8)	0.295	2.1 (-0.1-6.1)	5.1 (1.5-13.0)	0.234
Total IgE levels (UI/ml)	187	149.8 (42.1-434.5)	125.7 (39.3-449.3)	0.641	607.5 (210.3-1316.3)	212.0 (76.6-1223.0)	0.141	91.8 (27.2-210.4)	101.5 (24.1-220.4)	0.949
Absolute eosinophil count (cells/μl)	192	300.0 (200.0-500.0)	300.0 (100.0-500.0)	0.209	340.0 (255.0-695.0)	430.0 (165.0-545.0)	0.616	300.0 (200.0-400.0)	200.0 (100.0-400.0)	0.073
Eosinophil percentage (%)	186	4.3 (2.6-6.8)	3.7 (1.5-7.0)	0.190	7.0 (3.5-10.1)	5.4 (3.0-8.1)	0.134	3.8 (2.2-5.6)	2.6 (1.4-5.5)	0.151
Comorbidities										
Otorhinolaryngology disease	202	24 (23.8)	29 (28.7)	0.523	10 (33.3)	13 (31.7)	1.000	14 (19.7)	16 (26.7)	0.406
Gastroesophageal reflux	203	16 (15.7)	33 (32.7)	**0.005**	9 (29.0)	24 (57.1)	**0.020**	7 (9.9)	9 (15.2)	0.426
Sleep apnea	202	15 (15.0)	12 (11.8)	0.540	5 (16.7)	4 (9.3)	0.473	10 (14.3)	8 (13.6)	1.000
Obesity	197	37 (36.6)	33 (34.4)	0.768	9 (29.0)	12 (29.3)	0.796	28 (40.0)	21 (38.2)	0.856
Atopy	179	69 (76.7)	70 (78.7)	0.858	27 (87.1)	39 (90.7)	0.713	42 (71.2)	31 (67.4)	0.676
Other allergic phenotypes	202	72 (70.6)	74 (74.0)	0.639	28 (90.3)	37 (86.0)	0.726	44 (62.0)	37 (64.9)	0.853
Rhinitis	202	65 (63.7)	67 (67.0)	0.659	28 (90.3)	36 (83.7)	0.505	37 (52.1)	31 (54.4)	0.859
Dermatitis	202	18 (17.6)	20 (20.0)	0.721	11 (35.5)	14 (32.6)	0.808	7 (9.9)	6 (10.5)	1.000
Drug allergy	202	17 (16.7)	22 (22.0)	0.376	3 (9.7)	6 (14.0)	0.726	14 (19.7)	16 (28.1)	0.299
Food allergy	202	7 (6.9)	12 (12.0)	0.236	4 (12.9)	6 (14.0)	1.000	3 (4.2)	6 (10.5)	0.186
Age of asthma onset (years)	188	16.0 (5.0-40.0)	20.0 (6.0-44.8)	0.572	13.0 (5.0-24.0)	15.0 (6.0-28.8)	0.257	22.5 (12.0-44.5)	30.0 (6.8-50.0)	0.756
Family history										
Allergy	205	70 (68.6)	71 (68.9)	1.000	28 (90.3)	32 (74.4)	0.132	42 (59.2)	39 (65.0)	0.589
Asthma	205	44 (43.1)	46 (44.7)	0.888	17 (54.8)	20 (46.5)	0.639	27 (38.0)	26 (43.3)	0.594

Asthma exacerbations	205									
OCS use	205	0 (0)	70 (68.0)	NA	0 (0)	31 (72.1)	NA	0 (0)	39 (65.0)	NA
ER visits	205	0 (0)	93 (90.3)	NA	0 (0)	37 (86.0)	NA	0 (0)	56 (93.3)	NA
Hospitalizations	205	0 (0)	20 (19.4)	NA	0 (0)	8 (18.6)	NA	0 (0)	12 (20.0)	NA
Asthma severity	200									
Mild		8 (7.9)	0 (0)	**0.007**	6 (19.4)	0 (0)	**0.005**	2 (2.8)	0 (0)	0.500
Moderate		9 (8.9)	4 (4.0)	0.251	3 (9.7)	4 (9.3)	1.000	6 (8.5)	0 (0)	**0.032**
Severe		84 (83.2)	95 (96.0)	**0.005**	21 (67.7)	36 (83.7)	0.060	63 (88.7)	59 (100)	**0.008**
Asthma control	191									
Well controlled		55 (56.1)	26 (28.0)	**$1.3x10^{-4}$**	9 (31.0)	3 (7.7)	**0.022**	46 (66.7)	23 (42.6)	**0.010**
Partially controlled		29 (29.6)	27 (29.0)	1.000	11 (37.9)	12 (30.8)	0.609	18 (26.1)	15 (27.8)	0.841
Poorly controlled		14 (14.3)	40 (43.0)	**$1.2x10^{-5}$**	9 (31.0)	24 (61.5)	**0.016**	5 (7.2)	16 (29.6)	**0.001**
Pharmacological treatment										
SABA	205	47 (46.1)	79 (77.5)	**$6.5x10^{-6}$**	19 (61.3)	34 (79.1)	0.120	28 (39.4)	45 (76.3)	**$3.7x10^{-5}$**
ICS	205	95 (93.1)	103 (100)	**0.007**	26 (83.9)	43 (100)	**0.011**	69 (97.2)	60 (100)	0.500
LABA	205	90 (88.2)	101 (98.1)	**0.006**	25 (80.6)	42 (97.7)	**0.019**	65 (91.6)	59 (98.3)	0.124
LTRA	205	50 (49.0)	63 (61.8)	0.091	11 (35.5)	21 (48.8)	0.342	39 (54.9)	42 (71.2)	0.070
OCS	204	7 (6.9)	51 (50.5)	**$1.3x10^{-12}$**	2 (6.5)	23 (53.5)	**$1.6x10^{-5}$**	5 (7.0)	28 (48.3)	**$1.1x10^{-7}$**
SAMA	205	19 (18.6)	42 (41.2)	**$7.0x10^{-4}$**	0 (0)	5 (11.6)	0.070	19 (26.8)	37 (62.7)	**$4.4x10^{-5}$**
LAMA	205	25 (24.5)	31 (30.4)	0.433	2 (6.5)	6 (14.0)	0.455	23 (32.4)	25 (42.4)	0.276
Theophylline	205	0 (0)	9 (8.8)	**0.003**	0 (0)	1 (2.3)	1.000	0 (0)	8 (13.6)	**0.001**
Antihistamines	205	58 (56.9)	74 (72.5)	**0.028**	21 (67.7)	39 (90.7)	**0.017**	37 (52.1)	35 (59.3)	0.480
Azithromycin	205	5 (4.9)	15 (14.7)	**0.032**	2 (6.5)	7 (16.3)	0.288	3 (4.2)	5 (13.6)	0.066
Biological therapies	205	14 (13.7)	16 (15.7)	0.844	8 (25.8)	9 (20.9)	0.780	6 (8.5)	7 (11.9)	0.567
Immunotherapy	204	21 (20.6)	17 (16.8)	0.590	16 (51.6)	16 (37.2)	0.243	5 (7.0)	1 (1.7)	0.222
Antibiotics	205	18 (17.8)	45 (44.1)	**$6.8x10^{-5}$**	2 (6.5)	15 (34.9)	**0.005**	16 (22.9)	30 (50.8)	**0.002**
Medication adherence	203	25.0 (23.0-25.0)	25.0 (23.0-25.0)	0.121	24.0 (19.0-25.0)	23.5 (20.0-25.0)	0.570	25.0 (25.0-25.0)	25.0 (23.0-25.0)	0.444
Home environment (rural)	204	52 (51.0)	60 (58.8)	0.325	18 (58.1)	25 (59.5)	1.000	37 (52.1)	25 (41.7)	0.292
Household pets	201	48 (48.5)	49 (48.0)	1.000	15 (51.7)	20 (47.6)	0.811	33 (47.1)	29 (48.3)	1.000
Education level	204									
No schooling completed		2 (2.0)	1 (1.0)	1.000	0 (0)	1 (2.4)	1.000	2 (2.8)	0 (0)	0.500
Lower secondary education		40 (39.2)	39 (38.2)	1.000	4 (12.9)	11 (26.2)	0.243	36 (50.7)	28 (46.7)	0.726
Higher secondary education		37 (36.3)	41 (40.2)	0.666	15 (48.4)	19 (45.2)	0.816	22 (31.0)	22 (36.7)	0.578
Higher education		23 (22.5)	21 (20.6)	0.865	12 (38.7)	11 (26.2)	0.312	11 (15.5)	10 (16.7)	1.000

Descriptives are represented by the mean (standard deviation) and the median (interquartile range) values for continuous variables with a normal and non-normal distribution, respectively, and the count (proportion) for categorical variables. Differences between groups were evaluated using t-test and Mann-Whitney U-test for continuous variables with normal and non-normal distribution, respectively, and Fisher's exact test for categorical variables. Statistically significant differences are indicated in boldface (p<0.05).

Abbreviations: FEV_1: Forced expiratory volume in the first second; FVC: Forced vital capacity; BDR: Bronchodilator response; IgE: Immunoglobulin E; OCS: Oral corticosteroids; ER: Emergency room; SABA: Short-acting beta agonists; ICS: Inhaled corticosteroids; LABA: Long-acting beta agonists; LTRA: Leukotriene receptor antagonists; SAMA: Short-acting muscarinic antagonists; LAMA: Long-acting muscarinic antagonists; NA: Not applicable.
